# It’s never too early: How college students view aging and prepare for it

**DOI:** 10.1093/geroni/igaf122.3485

**Published:** 2025-12-31

**Authors:** Kelly O’Sullivan, Raven Weaver, Jill Juris, Cory Bolkan

**Affiliations:** Washington State University, Pullman, Washington, United States; Washington State University, Pullman, Washington, United States; Appalachian State University, Boone, North Carolina, United States; Washington State University, Vancouver, Washington, United States

## Abstract

As society ages rapidly, people must prepare for their own healthy aging trajectories. Perceptions of aging and expressions of ageism negatively influence aging experiences, contributing to poorer outcomes (e.g., chronic disease, decreased independence, earlier mortality). In contrast, aging preparedness (e.g., advanced care planning, financial security, retirement planning) leads to better outcomes, including increased autonomy and independence. This study examined how college students (N = 277, Mage=19.83; 66.1% White) perceived their own aging in relation to family, friends, religion, leisure, lifestyle, money, work, and health, and how these perceptions related to their preparedness for aging. We sought to answer two research questions: (1) how do college students perceive aging in these domains? and (2) how do perceptions of aging influence views of aging preparedness? Results indicated that students generally held positive aging perceptions, expressing optimism about anticipated later life experiences. Linear regression revealed significant associations between perceptions of aging preparedness in later life and perceptions of friends (p = 0.016), religion (p < 0.001), leisure (p = 0.008), lifestyle (p = 0.044), money (p = 0.010), and health (p < 0.001). These findings suggest that it is never too early for college students to prepare for aging. Students with positive perceptions of aging felt more prepared for older adulthood; thus, it is important to disrupt negative perceptions of aging early in life, which has the potential to improve health outcomes and foster greater autonomy in later years.

